# Multidrug-Resistant *Acinetobacter baumannii*: An Emerging Health Threat in Aseer Region, Kingdom of Saudi Arabia

**DOI:** 10.1155/2018/9182747

**Published:** 2018-02-04

**Authors:** Mohammed K. Almaghrabi, Martin R. P. Joseph, Mohammed M. Assiry, Mohamed E. Hamid

**Affiliations:** ^1^Department of Microbiology and Clinical Parasitology, King Khalid University, Abha, Saudi Arabia; ^2^Microbiology Laboratories, Aseer Central Hospital, Abha, Saudi Arabia

## Abstract

**Objective:**

The study aims to determine the prevalence of multidrug-resistant *A. baumannii* in Aseer Region, Kingdom of Saudi Arabia.

**Methods:**

This study evaluated the antibiotic susceptibility of ninety-four (*n* = 94) clinical isolates of *A. baumannii*. The isolates were collected from the south region of Saudi Arabia, and notably Aseer Region, during the period from 15 October 2014 to 15 January 2015. The isolates were tentatively identified as *A. baumannii* by routine bench tests and were confirmed by using VITEK® 2 Compact. The latest instrument was used to identify antibiotic susceptibility of these isolates.

**Results:**

Antibiotic susceptibility in this study showed that 69% of these isolates were multidrug-resistant strains. Moreover, they were highly resistant to carbapenem drugs. Several strains of these isolates were found to be extremely resistant to test antibiotics and were only sensitive to one or two of them.

**Conclusion:**

High rate of multidrug-resistant *A. baumannii* bacteraemia has emerged in the south region of Saudi Arabia as an important health problem. Therefore, it is considered as a new threat in hospitals, which requires a tremendous effort to stop its escalation and spread.

## 1. Introduction

*Acinetobacter baumannii* is a strictly aerobic, nonmotile, Gram-negative, nonfermentative, oxidase-negative, and catalase-positive organism. Within the healthcare settings, *Acinetobacter baumannii* has become a common pathogen, which can infect the respiratory tract, blood, soft tissues, and urinary tract of an individual. It is the etiological agent of nosocomial infections resulting in septicemia, meningitis, endocarditis, pneumonia, wound, and urinary tract infections [1, 2]. There are 32 *Acinetobacter* named and unnamed species, which have been identified [3]. The *Acinetobacter* species cause infections, which are associated with increased morbidity and mortality rates [4, 5].

*A. baumannii* is tolerant to wide ranges of temperature, pH, and humidity. Studies have shown that this bacterium can survive on dry surfaces for 5 months, posing a challenge to hospital infection control measures [4]; therefore, this pathogen is considered as progressively important nosocomial pathogen, which can cause outbreak of serious infections. Despite the fact that the organism is often nosocomial, initial infection can be transmitted by patients, admitted from other hospitals [6, 7].

Almost 25 years ago, *A. baumannii* was found to be resistant against antimicrobial drugs, such as aminopenicillins, cephalosporins, first- and second-generation cephalosporins, cephamycins, aminoglycosides, ureidopenicillins, chloramphenicol, and tetracyclines. Strains of *A. baumannii* have started to acquire resistance to newly developed antimicrobial drugs and become prevalent in many hospitals [8]. More recently, the term “extensively drug-resistant” *A. baumannii* (XDRAB) has been used to characterize bacterial isolates resistant to all authorized antibiotics except two categories of antibiotic such as tigecycline and polymyxins [9].

The possibility of *A. baumannii* isolation from hospitalized patients is related to some important factors, such as bacterial colonization, medical staff-to-patient ratio, and other ward characteristics [10]. The current study was designed to determine the prevalence of multidrug-resistant *A. baumannii* in Aseer Region. Bacterial strains were isolated from patients who attended Aseer Central Hospital, Aseer Region, Kingdom of Saudi Arabia.

## 2. Materials and Methods

### 2. 1. Ethical Approval

This research was approved by Research Ethics Committee, College of Medicine, King Khalid University, Abha, Saudi Arabia.

### 2.2. Bacterial Isolates

**Figure 1:**
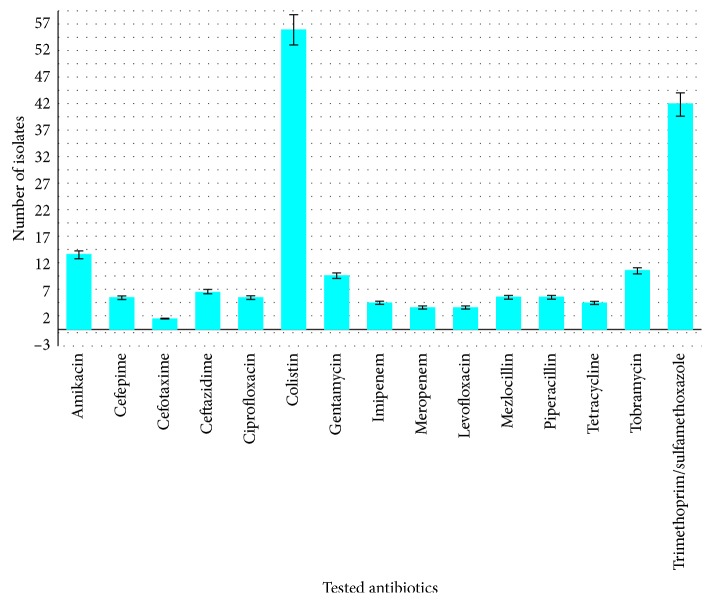
Multidrug resistance pattern of *Acinetobacter baumannii*.

94 clinical isolates of *A. baumannii* were collected from the south region of Saudi Arabia and notably Aseer Region (Figure 1) during the period from 15 October 2014 to 15 January 2015. All these strains were initially isolated and identified in Aseer Central Hospital laboratory and were sent to Department of Microbiology, College of Medicine, King Khalid University, for further assessment.

### 2.3. Media and Reagents

Two culture media were used in this experiment: blood agar (Oxoid Ltd., Basingstoke, and Hampshire, United Kingdom) and MacConkey agar (Oxoid). MacConkey agar was used as a selective and differential medium; while, blood agar was used for other processes, such as the sensitivity test. Biochemical reagents, such as catalase, oxidase, and indole, were used as initial identification tests.

### 2.4. Bacterial Identification and Purification

All clinical isolates were cultured on blood and MacConkey agar and then tested for Gram staining to identify Gram reaction and bacterial morphology. In order to ensure that all isolates are pure, they were cultured several times. Several biochemical tests were undertaken to confirm that all these isolates were belonging to *A. baumannii*, such as catalase, oxidase, and indole tests. Identification of all strains was done, using the automated VITEK 2 system (bioMérieux) following manufacturer instructions as briefly described below.

### 2.5. Antibiotic Susceptibility of *A. baumannii*

All isolates were tested for antibiotic susceptibility, using the automated VITEK 2 Compact system. All samples were cultured on blood agar, and then, a suspension was made for every isolate. Liquid suspension of all isolates was loaded on the VITEK system and left overnight to get the results. The next day, the results have illustrated the identification and antibiotic susceptibility of loaded samples.

VITEK 2 system was used for authenticating names of *Acinetobacter* species as described by the manufacturer (bioMérieux Inc., Durham, NC 27712, USA). The VITEK card contains 64 wells, which hold different fluorescent biochemical assays. Out of the 64, 20 were carbohydrate assimilation; 4 were phosphatase, urea, nitrate, and actidione tests. The VITEK 2 machine controlled the card automatically including the filling, sealing, and then transferring the cards into the linked incubator (35°C). Each output report is decoded according to a particular algorithmic system. The acquired results were compared to the ID-GN (identification of Gram-negative bacteria) databank. In case of most known *Acinetobacter* species with a clear cut profile, the system led to a correct identification of the unknown organism.

## 3. Results

### 3.1. Identification of Tested Isolates

Colony characteristics of isolated colonies were obtained using blood and MacConkey agar. On MacConkey agar, colonies of *A. baumannii* appeared as a nonlactose fermenter. On blood agar, colonies of all isolates were appeared as nonpigmented, domed, and mucoid, with smooth-to-pitted surfaces in a diameter of 1-2 mm colonies. All strains of *A. baumannii* were found to be catalase positive and oxidase/indole negative. The isolates were tentatively identified as *A. baumannii* by routine bench tests and have been confirmed by using VITEK 2 Compact.

### 3.2. Antibiotic Susceptibility of *A. baumannii*

**Figure 2:**
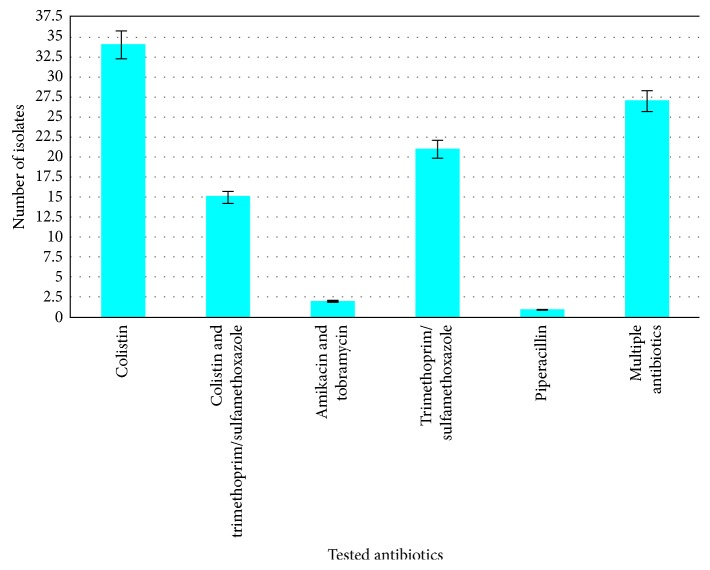
Numbers of sensitive *Acinetobacter baumannii* isolates to tested antibiotics.

Antibiotic susceptibility testing has shown that 69% of these isolates were multidrug-resistant strains (Figure 1). 34 isolates were resistant to all antibiotics of interest and were only sensitive to colistin. The results have also shown that 15 of these isolates were only sensitive to colistin and a combination of trimethoprim and sulfamethoxazole, whereas 21 of isolates were sensitive to the combination of trimethoprim and sulfamethoxazole. One isolate and other two of them were resistant to all tested antibiotics except piperacillin and amikacin and tobramycin, respectively. The correlation between the number of isolates and their sensitivity to all tested antibiotics is shown in Figure 2.

## 4. Discussion

Multidrug-resistant *A. baumannii* is currently a major cause of respiratory infections in intensive care unit patients. However, *Acinetobacter* was identified as a low-virulent pathogen, which infects mostly immunocompromised hosts; however, the emergence of resistant strains to antibiotics over the past several years has increased. Therefore, the concern toward this phenomenon has increased as well. MDR *A. baumannii* is recognized as one of the most difficult nosocomial pathogens to be treated and controlled [11, 12].

In this study, multidrug-resistant and extensively resistant *A. baumannii* strains have been isolated and identified in the southern region of Saudi Arabia. The study has shown that 74% of these isolates are multidrug-resistant strains. Among these MDR strains, approximately 50% are extensively drug-resistant isolates, which are sensitive to colistin and resistant to all other drugs of choice. This should raise the concern toward this potentially dangerous pathogen. The most effective antibiotic with high spectrum to treat *A. baumannii* is colistin (60%) and then the combination of trimethoprim and sulfamethoxazole (46%); these rates of spectrums were approximately compatible with previous studies [13].

*A. baumannii* isolates were extremely resistant to two carbapenem drugs: imipenem and meropenem. Among tested strains, only 5 (0.05%) and 4 (0.04%) isolates were susceptible to imipenem and meropenem, respectively. This process indicated that these isolates were more dangerous than previously identified MDR strains, which showed more than 90% susceptibility to both drugs [8].

Susceptibility of *A. baumannii* to carbapenems showed a clear drop, and it denotes a main epidemiological worry. The resistance mechanism was found due to class D OXA-type enzymes (oxa-23 and oxa-24/40) with carbapenemase activity [14]. This fact made the medical choices very limited mainly based on polymyxin combinations with other antibiotics. Carbapenem-resistant *Acinetobacter* spp. have been described from many parts of the world, and Saudi Arabia is not an exception [15]. These authors examined 64 *Acinetobacter baumannii* isolates during 2013 and 2014 from four different medical centers in Saudi Arabia and Egypt. All the isolates were found to be resistant to ceftazidime and ciprofloxacin. They reported the emergence of ST236 in Saudi Arabia and Egypt and the spread of distinct carbapenem-resistant *A. baumannii* clones belonging to ST884, ST945, and ST1096 in Saudi Arabia [15]. Another study revealed a detailed molecular analysis of many isolates from Saudi Arabia including the detection of OXA-58 gene in five isolates and other OXA genes [16]. A number of studies must be undertaken to eliminate extra emergence and distribution of this potent pathogen across the country. Some molecular projects are being processed and designed to study the type of antibiotic-resistant genes in these strains.

## 5. Conclusion

Higher rate of multidrug-resistant *A. baumannii* bacteraemia has emerged in the southern region of Saudi Arabia as an important health issue. Such a higher prevalence has now been considered as a new threat in the clinical settings, which requires a tremendous effort to stop its escalation and spread. The occurrence of *Acinetobacter* strains and the antimicrobial resistance degree continue to rise. The results among the patients, exposed to infections from these organisms, were discovered to be strong enough. *Acinetobacter* strains also indicated vast challenges for the healthcare facilities, public health, and elderly population.
